# The ileal microbiome and mucosal immune profiles in response to dietary supplementation of ultra-grinded *Astragalus membranaceus* in weaned goats

**DOI:** 10.3389/fmicb.2023.1309520

**Published:** 2023-12-21

**Authors:** Guowang Luo, Kefyalew Gebeyew, Chuanshe Zhou, Zhiliang Tan, Wenzhu Yang, Dongyan Niu, Tao Ran, Yong Liu

**Affiliations:** ^1^State Key Laboratory of Herbage Improvement and Grassland Agro-ecosystems, Ministry of Agriculture and Rural Affairs, College of Pastoral Agriculture Science and Technology, Lanzhou University, Lanzhou, Gansu, China; ^2^CAS Key Laboratory for Agro-Ecological Processes in Subtropical Region, National Engineering Laboratory for Pollution Control and Waste Utilization in Livestock and Poultry Production, and Hunan Provincial Key Laboratory of Animal Nutritional Physiology and Metabolic Process, Institute of Subtropical Agriculture, the Chinese Academy of Sciences, Changsha, Hunan, China; ^3^University of Chinese Academy of Sciences, Beijing, China; ^4^Lethbridge Research and Development Centre, Lethbridge, AB, Canada; ^5^Faculty of Veterinary Medicine, University of Calgary, Calgary, AB, Canada

**Keywords:** microbial community, ileal mucosal immune, dietary supplementation, ultragrinded *Astragalus membranaceus*, weaned goats

## Abstract

Weaning goats are susceptible to diarrhea and have weakened immune functions due to physiological, dietary and environmental stresses. *Astragalus membranaceus* (*A. membranaceus*), a traditional Chinese medicinal herb, has been shown to improve growth performance and immunity in weaned ruminants. However, the influence mechanism of *A. membranaceus* on intestinal microbiota and mucosal immunity in weaned goats is still unknown. This study investigated the effects of ultra-grinded *A. membranaceus* (UGAM) on the immune function and microbial community in the ileum of weaned goats. Eighteen healthy weaned Xiangdong black goats (BW, 5.30 ± 1.388 kg) were used in a study of completely randomized block design with 28 days long. The animals were randomly assigned to either a basal diet supplemented with 10 g/d of milk replacer (CON, *n* = 9) or the CON diet supplemented with 10 g/head UGAM (UGAM, *n* = 9). Supplementation of UGAM increased (*p* < 0.05) the plasma concentrations of total protein and albumin. Meanwhile, the addition of UGAM reduced (*p* < 0.05) the relative mRNA expression of the *IL-6* gene (a marker of inflammation), indicating the potential immunomodulatory effect of UGAM. Moreover, the relative abundances of *Verrucomicrobiota* and *Mycoplasma* were lower (*p* < 0.05) in the ileum of goats supplemented with UGAM than CON. These findings suggest that dietary supplementation of UGAM may have enhanced the ileum health of weaned goats by reducing inflammation factor expression and reducing the relative abundance of pathogenic microbes. The observed beneficial effects of ultra-grinded *A. membranaceus* on ileal mucosal immune and the community of ileal microbiota indicate its potential to be used as a viable option for promoting the well-being of weaned goats under weaning stress.

## 1 Introduction

Weaning is a critical stage in the development of goats, during which they undergo dietary transition, and social and environmental changes (Zobel et al., 2020). This is the most stressful period of animal life that is often associated with challenges such as diarrhea and immune suppression, which can have detrimental effects on the health and growth performance of weaned goats (Chen et al., 2020). It is reported that the small intestine barrier function of piglets decreases rapidly shortly after weaning, accompanied with reduced absorptive capacity (Wijtten et al., 2011). This is associated with the disruption of tight junction structures and reduced mucin production, which ultimately leads to increased intestinal permeability (Wang et al., 2016). For young ruminants like goats, weaning signifies the cessation of their capacity to obtain immunoglobulins, glutathione peroxidase, lysozymes, and other components from maternal milk (Huang et al., 2023; Yuan et al., 2023), thus leading to a decrease in acquired immune barrier function (Zhang et al., 2016). Moreover, it is worth noting that weaning has the potential to cause oxidative damage in the intestine of goats (Chen et al., 2020). Considerable effort has been made toward developing various nutritional and management strategies to improve animal immunity and health (Zheng et al., 2020). The use of traditional herbal medicines has been one of the most studied areas during the last decades.

*Astragalus membranaceus* (*A. membranaceus*) root powder, commonly in therapeutic use for both humans and livestock, is now gaining increasing interest in being used as a feed additive. It contains lots of bioactive compounds, including polysaccharides (Peng et al., 2023), flavonoids (Liang et al., 2020), and saponins (Ionkova et al., 2014). Therefore, it possesses various beneficial effects such as anti-apoptosis (Liao et al., 2023), anti-inflammatory (Adesso et al., 2018), immunomodulatory (Du et al., 2022) and neuroprotective properties (Gao et al., 2022; Li et al., 2022). These properties make it a promising candidate for improving weaned goats’ health and immune response. Several studies have demonstrated the positive impact of *A. membranaceus* supplementation on growth performance and immune function in weaned ruminants. For instance, a study demonstrated that *A. membranaceus* enhanced growth performance, rumen fermentation, and immune response of Tibetan sheep (Wang et al., 2021). Another study reported an improvement in the antioxidant capacity and stability of goat muscle by reducing myoglobin oxidation and lipid oxidation levels, and increasing antioxidant enzyme activity with supplementation of *A. membranaceus* root (Luo et al., 2020). However, the specific effects of *A. membranaceus* on the microbiota composition, tight junction, and immune functions of the ileum of weaned goats and its underlying mechanisms are still not fully understood.

Ultra-fine grinding technology that uses mechanical or hydrodynamic methods to crush material into fine particles ranging from 10 to 15 μm (Chaumeil, 1998), is widely used in processing Chinese herbs. Compared to the conventional grinding method, the ultra-fine ground herbs showed better dissolution rate, dispersion, utilization rate, and efficacy when applied to animals (Xu et al., 2015; Du et al., 2020). Similarly, ultra-grinding technology is involved in processing astragalus root, and the water-holding capacity and polysaccharide solubility increased as the particle sizes of astragalus root powder decreased from 300 to 7.56 μm (Li et al., 2011). Moreover, dietary supplementation of *A. membranaceus* powder at the concentration of 5 g/kg of diet enhanced serum antioxidant status, and its efficacy linearly increased as the *A. membranaceus* particle size decreased from 300 to 37 μm in broilers (Zhang et al., 2013). Given that the ileum represents a site for nutrient absorption and intestinal microbial and immune function of the small intestine, we hypothesized that supplementation of ultra-grinded *A. membranaceus* (UGAM) powder might improve the microbiota composition, tight junction, and immune functions of the ileum. This study aimed to assess the effects of feeding a basal diet supplemented with UGAM on the blood metabolites, and immune function, tight junction and microbiota composition in the ileum of weaned goats.

## 2 Materials and methods

### 2.1 Animal experimental design and diets

Eighteen healthy weaned Xiangdong black goats (42 days old) with similar initial body weight (BW; 5.30 ± 1.388 kg) were blocked by BW and randomly allocated into one of two treatments (*n* = 9/treatment). The experiment was a completed randomized block design with two treatments that were a basal diet supplemented with 10 g/d of milk replacer (CON) and the CON diet supplemented with 10 g/d ultra-grinded *Astragalus membranaceus* (UGAM) for each goat. The UGAM was prepared using the root of *A. membranaceus*, which was purchased locally, chopped, dried overnight at 60°C, and then ultra-ground into micron order (D50 = 14.74 μm, D90 = 52.06 μm) using a Chinese medicine ultrafine grinding machine (WZJ-6B, Jinan Beili Powder Engineering Technology, Jinan, China). The dose of UGAM used in the present study was based on a previous report by Hao et al. (2020). The milk replacer was supplied by the Beijing Precision Animal Nutrition Research Center (Q/HDJZA 0004-2018, Beijing, China). The ingredients and nutrient contents of the experimental diet were detailed in Table 1. The experimental period consisted of a pre-trial period of 7 days and an experimental period of 28 days. Goats were housed and fed in individual cages and had free access to clean water throughout the experimental period.

**Table 1:** Ingredients and nutrient levels of the basal experimental diet.

Ingredients	Diet (g/kg DM)	Chemical composition^2^	
Extruded-soybean	244	DM, g/kg	952.9
Corn grain	220	CP, g/kg	204.6
Whey powder	150	Crude fat, g/kg	38.8
Fatty powder	50	ADF, g/kg	189.0
Calcium carbonate	5	NDF, g/kg	279.0
Calcium hydro-phosphate	15	Calcium, g/kg	2.1
Salt	6	Phosphate, g/kg	9.3
Premix^1^	10	ME, Mcal/kg	2.88
Alfalfa hay	300		

^1^The premix consisted of (per kilogram of premixed material): 2.5 g FeSO_4_·7H_2_O, 0.8 g CuSO_4_·5H_2_O, 3 g MnSO_4_·H_2_O, 10 mg Na_2_SeO_3_, 40 mg KI, 30 mg CoCl_2_·6H_2_O, 95,000 IU vitamin A, 17,500 IU vitamin D, 18,000 IU vitamin E. ^2^DM, dry matter; CP, crude protein; ADF, acid detergent fiber; NDF, neutral detergent fiber. The metabolizable energy (ME) was estimated (Sung and Kim 2020).

### 2.2 Blood sampling and organ collection

#### 2.2.1 Blood biochemical indices analysis

Before morning feeding, blood was sampled from each goat at the end of the experimental period (78 days old). Each goat was subjected to neck venipuncture using disposable needles, and about 4 mL of blood was collected into anticoagulant tubes containing EDTA-K (Changsha Yiqun Medical Equipment Co., Ltd., Changsha, China). The samples were placed at room temperature for 10 min, and then centrifuged at 3,000 × g for 20 min at 4°C to obtain plasma. The obtained plasma samples were stored at −20°C until the analysis.

Blood biochemical indices were determined using commercial assay kits (Nanjing Jiancheng, Nanjing, China) and a spectrophotometer (Barloworld Scientific Ltd., Genova, Italy) in CAS Key Laboratory for Agro-Ecological Processes in Subtropical Region. The measured blood indices included total protein (TP; kit #03183734190), albumin (ALB; kit #03183688122), alanine aminotransferase (ALT; kit #20764957322), aspartate aminotransferase (AST; kit #20764949322), alkaline phosphatase (ALP; kit #03333701190), gamma-glutamyl transferase (GGT; kit #03002721122), lactate dehydrogenase (LDH; kit #03004732122), blood urea nitrogen (BUN; kit #04460715190), glucose (GLU; kit #11929992216), triglycerides (TG; kit #20767107322), total cholesterol (CHOL; kit #03039773190), amylase (AMS; kit # 03183742122), low-density lipoprotein cholesterol C3 (LDL-C3; kit #03038866322), C-reactive protein 3 (CRPL3; kit #03263991190), amyloid precursor protein (Amy-p; kit #20766623322), total bilirubin 3 (BILT3; kit #03146022122), lactic acid (LACT; kit #03183700190), blood ammonia (NH_3_L; kit #20766682322). All measurements were carried out according to the instructions of the supplier.

#### 2.2.2 Organ index

On day 79, all goats were euthanized and slaughtered after 12 h of fasting and depriving of water. The organs, including the heart, kidney, adrenal gland, thymus, pancreas, thyroid, spleen, and lymph nodes from the jejunum, ileum, and colon, were isolated, collected, and weighed. The organ index was calculated as the weight of each organ divided by the final live weight before slaughter.

### 2.3 Ileal immune function, tight junction, and microbiota composition

#### 2.3.1 Ileal mucosa and contents sample collection

Approximately 15 mL of ileal contents were collected from the central part of the ileum into a 20 mL sterile centrifuge tube, snap-frozen in liquid nitrogen, and stored at −80°C for further analysis. Then, about 10 cm of middle ileum was taken and washed with cold PBS, and 2 ~ 4 cm of ileum tissue was cut and fixed in 10% polyoxymethylene-phosphoric acid buffer for morphological measurement. The ileal mucosa sample was collected from the rest ileal tissue and stored at −80°C to measure the expression of mucosal immune and tight junction-related genes.

#### 2.3.2 Real-time PCR for mucosal immune- and tight junction-related genes

Isolation of RNA and cDNA preparation from the collected ileal mucosa samples was carried out according to Ran et al. (2016). Expression levels of target genes related to immune and barrier functions in ileal tissue were determined by real-time PCR, which was performed on an ABI-7900HT qPCR system (Applied Biosystems, Foster City, United States) using FG Power SYBR Green PCR Master Mix (Applied Biosystems). Primer sequences for the target and internal reference genes are presented in Table 2. A melt curve analysis was performed for each gene at the end of the PCR run. The expression level for each gene was calculated after its cycle threshold (Ct) was normalized to the Ct for *β-actin* using the 2−^ΔΔCt^ method (Livak and Schmittgen, 2001).

**Table 2:** Primers used for real-time PCR analysis of genes related to intestinal immune and barrier functions.

Gene name	GenBank accession number	Primer sequences (5′-3′)^1^	Product length (bp)
*CLD1*	102174598	F: GGAGGATGGGCGGTGATAGA	164
		R: GTCTGTGCCAATTGAGGCTG	
*TJP1*	102178667	F: GGACAAAGAGAAGGGTGAGACC	132
		R: GCAAAAGACCAACCGTCAGG	
*TJP2*	102180039	F: CCTTGTGAGTGGGATTGGCA	104
		R: ATGCCTGGAGCCTGGTAAAG	
*TJP3*	102174294	F: TTTTGACTGTTTCTAGGCCCCC	147
		R: GCGGGGGTCCTTGCTGA	
*IL-1β*	100860816	F: CCTCCGATGAGCTTCTGTGT	149
		R: GAACACCACTTCTCGGCTCA	
*TNF-α*	100861232	F: GCCTCAGCCTCTTCTCCTTC	100
		R: GGGGACTGCTCTTCCCTCT	
*IL-6*	102172767	F: TCCTTCACACGTACTGCACC	172
		R: AAAACTGGCCGTGTTGCTTC	
*IL-17*	102171111	F: TGACATTGTGGCTCACCCTC	197
		R: CCGGGTGATGTTGTAATCCCA	
*TLR4*	100860955	F: GACCCTTGCGTCCAGGTTG	151
		R: ACCTGGAGAAGTTATGGCTGC	
*EGFR*	102172767	F: CTGCACCATCGACGTCTACA	147
		R: ATTCTCTCGTCCCCCTGGAT	
*MUC2*	102175111	F: CTGAAGCCCGGAGACATGAA	190
		R: CATTGCGAGGGATGCACTTC	
*OCLN*	102185782	F: TCGCTGAGAGAAGATTGGCT	187
		R: CGAACGTGCATCTCTCCACT	
*F11R*	102175451	F: TGGGTACGAAGGCGAAAGTC	143
		R: GGACAGCTTGGCAGGGTTAT	
*FOXP3*	102188755	F: CAGCACCCTTTTGACTGTGC	116
		R: GGCAGTGCTTGAGGAAGTCT	
*TGFB1*	102191364	F: TGGCTGACCCACAGAGAGGA	100
		R: ACCCTGCGTTAATGTCCACTT	

^1^F, forward; R, reverse.

#### 2.3.3 Genomic DNA extraction and library preparation

The ileal content samples were thawed at 4°C overnight, and the DNA of ileal microbiota was extracted with the T Guide S96 Magnetic Soil/Stool DNA Kit (Tiangen Biotech Co. Ltd., Beijing, China) according to the manufacturer’s instructions. The integrity and concentration of extracted DNA were checked using agarose gel electrophoresis and Qubit dsDNA HS Assay Kit and Qubit 4.0 Fluorometer (Invitrogen, Thermo Fisher Scientific, Oregan, United States), respectively. The extracted DNA was stored at −20°C until sequencing.

The universal primer 27F/1492R (Forward: AGRGTTTGATYNTGGCTCAG; Reverse: TASGGHTACCTTGT-TASGACTT) set was used to amplify the full-length of bacterial 16S rRNA gene from the extracted genomic DNA (Frank et al., 2008). Both the forward and reverse primers were tailed with sample-specific PacBio barcode sequences to allow for multiplexed sequencing, which reduces chimera formation as compared to the alternative protocol in which primers are added in the second PCR reaction. Twenty-five cycles of PCR amplification were performed using the KOD One PCR Master Mix (TOYOBO Life Science), with initial denaturation at 95°C for 2 min, followed by 25 cycles of denaturation at 98°C for 10 s, annealing at 55°C for 30 s, and extension at 72°C for 1 min 30 s, and a final step at 72°C for 2 min. The amplicons were verified and purified using the 2% agarose gel electrophoresis and Agencourt AMPure XP Beads (Beckman Coulter, Indianapolis, United States), respectively, and then quantified using the Qubit 4.0 Fluorometer (Invitrogen, Waltham, United States). After the individual quantification step, amplicons were pooled in equal amounts to construct pair-end libraries. SMRT bell libraries were prepared from the amplified DNA by SMRT bell Express Template Prep Kit 2.0 according to the manufacturer’s instructions (Pacific Biosciences). Purified SMRT bell libraries from the pooled and barcoded samples were sequenced on a single PacBio Sequel II 8 M cell using the Sequel II Sequencing Kit 2.0.

#### 2.3.4 Bioinformatics analysis

The bioinformatics analysis of obtained raw data was performed on the BMK Cloud (Biomarker Technologies Co., Ltd., Beijing, China). Briefly, the raw reads were filtered and demultiplexed using the SMRT Link software (version 8.0) with the min Passes ≥ 5 and min Predicted Accuracy ≥ 0.9 to obtain the circular consensus sequencing (CCS) reads. Subsequently, the LIMA (version 1.7.0) was employed to assign the CCS sequences to the corresponding samples based on their barcodes. The CCS reads containing no primers, and those reads beyond the length range (1,200–1,650 bp) were discarded through the recognition of primers and quality filtering using the Cutadapt quality control process (version 2.7). The chimera sequences were detected and removed using the UCHIME algorithm (version 8.1) to obtain clean reads. Sequences with similarity ≥ 97% were clustered into the same operational taxonomic unit (OTU) using the USEARCH (version 10.0; Edgar, 2013), and the OTUs with abundance < 0.005% were filtered. Taxonomy annotation of the OTUs was performed based on the Naive Bayes classifier in QIIME2 (Bolyen et al., 2019) using the SILVA database (Quast et al., 2012). The α-diversity indices were calculated and displayed by the QIIME2 and R software, respectively. Beta diversity analysis was assessed using principal coordinate analysis (PCoA) and nonmetric multidimensional scaling (NMDS). Linear discriminant analysis (LDA) effect size (LEfSe) was applied to show the difference in the bacterial communities between the two treatments using the non-parametric factorial Kruskal-Wallis test with an alpha value of 0.05 and LDA score of 2.0. Finally, the PICRUSt2 software was used to predict the metagenomic metabolic function based on normalized OTU abundance and the KEGG pathway on level 2 (Langille et al., 2013; Comeau et al., 2017).

### 2.4 Statistical analysis

Data analysis for blood biochemistry indices, and expression of immune-related genes in the ileum were performed using IBM SPSS Statistics 26. T-test was used to compare the differences between the two treatment means. Wilcoxon rank-sum test was used to detect differential abundances of phyla and genera between the two treatments. Unless otherwise stated, treatment effects were considered significant at *p* ≤ 0.05, and tendencies were declared at 0.05 < *p* ≤ 0.10. The correlations between bacterial genera with genes related to immune and tight junction and plasm biochemical index were performed using Spearman’s rank correlation coefficient in the R package, and the results were presented in the form of a heatmap.

## 3 Results

### 3.1 Effects on blood biochemical indices

The results of blood biochemical indices are presented in Table 3. The supplementation of UGAM had a greater concentration of TP (*p =* 0.02) and ALB (*p =* 0.03) compared with the CON group. However, the other blood biochemical indices did not differ between CON and UGAM treatments, and all of them were within the normal physiologic range in weaned goats.

**Table 3:** Effects of UGAM supplementation on the blood biochemical indices of weaned goats.

Items^1^	Treatments^2^		SEM	*p*-value
	CON	UGAM		
TP, g/L	68.02	75.42	1.68	0.02
ALB, g/L	33.36	36.23	0.70	0.03
BUN, mmol/L	8.39	7.88	0.40	0.54
GLU, mmol/L	2.66	3.13	0.30	0.44
TG, mmol/L	0.57	0.47	0.03	0.14
CHOL, mmol/L	2.45	2.75	0.12	0.22
LDL-C3, mmol/L	0.95	1.16	0.08	0.21
CRPL3, mg/L	4.16	4.17	0.01	0.58
BILT3, μmol/L	3.80	4.07	0.16	0.41
LACT, mmol/L	6.04	7.03	1.08	0.66
NH_3_L, μmol/L	257.28	295.11	24.87	0.46
ALT, U/L	21.74	22.76	1.36	0.72
AST, U/L	96.89	95.44	6.20	0.91
ALP, U/L	284.11	259.22	33.45	0.72
GGT, U/L	41.11	38.00	2.04	0.46
LDH, U/L	386.00	429.00	24.96	0.40
AMS, U/L	27.56	22.00	1.88	0.14
amy-p, U/L	26.74	20.94	1.88	0.13

^1^TP, total protein; ALB, albumin; BUN, blood urea nitrogen; GLU, glucose; TG, triglycerides; CHOL, cholesterol; LDL-C3, low-density lipoprotein cholesterol C3; CRPL3, C-reactive protein 3; BILT3, bilirubin 3; LACT, lactic acid; NH_3_L, ammonia; ALT, alanine aminotransferase; AST, aspartate aminotransferase; ALP, alkaline phosphatase; GGT, gamma-glutamyl transferase; LDH, lactate dehydrogenase; AMS, amylase; amy-p, amyloid precursor protein. ^2^CON, the control group; UGAM, the ultra-grinded Astragalus membranaceus powder group.

### 3.2 Effects on organ index

The results for the organ index of weaned goats are presented in Table 4. Most of the organ index did not differ between the CON and UGAM treatments, except that the goats received the UGAM supplementation tended to have greater (*p* = 0.067) lung index as compared to the CON treatment. The indexes of organs related to immune function were similar between the CON and UGAM treatments.

**Table 4:** Effects of UGAM supplementation on the organ index of weaned goats.

Organ indices^1^, %	Treatments^2^		SEM	*P*-value
	CON	UGAM		
Heart	1.15	1.08	0.034	0.286
Liver	5.47	5.39	0.136	0.779
Spleen	0.37	0.50	0.044	0.176
Lung	3.22	4.19	0.268	0.067
Kidney	1.17	1.29	0.039	0.134
Adrenal grand	0.03	0.03	0.002	0.487
Thymus	0.41	0.41	0.045	0.995
Pancreas	0.39	0.39	0.017	0.996
Jejunal lymph nodes	0.09	0.09	0.012	0.953
Ileal lymph nodes	0.4	0.38	0.045	0.843
Colonic lymph nodes	0.13	0.25	0.050	0.217
Thyroid	0.55	0.58	0.032	0.623

^1^Organ index (%) = organ weight (g)/live weight (g) × 100; ^2^CON, the control group; UGAM, the ultra-grinded Astragalus membranaceus powder group.

### 3.3 Effects on the mRNA expression of genes related to ileal immune and barrier function

Dietary supplementation of UGAM did not affect the mRNA expression of most of the immune- and barrier function-related (mainly tight junction proteins, TJP) genes in the ileum, except *IL-6*, *TJP3,* and *Ocludin* (*OCLN*; Table 5). Among which, the relative expressions of *IL-6* (*p* < 0.05), and *OCLN* (*p* < 0.01) genes were lower in the UGAM treatment, whereas the relative expression of *TJP3* was greater (*p* < 0.05) with UGAM than CON treatment.

**Table 5:** Effects of UGAM supplementation on the relative expression of immune and tight junction-related genes in the ileum.

Genes^1^	Treatments^2^		SEM	*P*-value
	CON	UGAM		
*IL-1β*	0.86	0.62	0.115	0.303
*IL-6*	0.9	0.38	0.109	0.015
*IL-17*	0.93	0.93	0.090	0.976
*TNF-α*	1.04	0.71	0.146	0.274
*TGF-β1*	1.07	1.08	0.081	0.922
*TLR4*	0.99	1.07	0.085	0.650
*NF-κB*	1.16	1.37	0.108	0.348
*FOXP3*	1.01	1.34	0.089	0.068
*F_11_R*	1.02	1.23	0.114	0.363
*EGFR*	1.02	1.06	0.042	0.658
*MUC2*	0.93	0.71	0.106	0.319
*OCLN*	1.09	0.6	0.095	0.005
*CLD1*	0.93	0.88	0.096	0.798
*TJP1*	0.94	0.78	0.107	0.495
*TJP2*	0.88	0.71	0.106	0.448
*TJP3*	1.05	1.42	0.092	0.039

^1^IL-1β, interleukin-1β; IL-6, interleukin-6; IL-17, interleukin-17; TNF-α, tumor necrosis factor-α; TGF-β1, transforming growth factor-β1; TLR4, Toll-like receptor 4; NF-κB, nuclear factor kappa-B; FOXP3, forkhead box protein P3; F_11_R, F_11_ receptor; EGFR, epidermal growth factor receptor; MUC2, mucin-2; OCLN, occludin; CLD1, claudin-1; TJP1, tight junction protein 1; TJP2, tight junction protein 2; TJP3, tight junction protein 3; ^2^CON, the control group; UGAM, the ultra-grinded Astragalus membranaceus powder group.

### 3.4 Effects on the ileal microbial community

**Figure 1:**
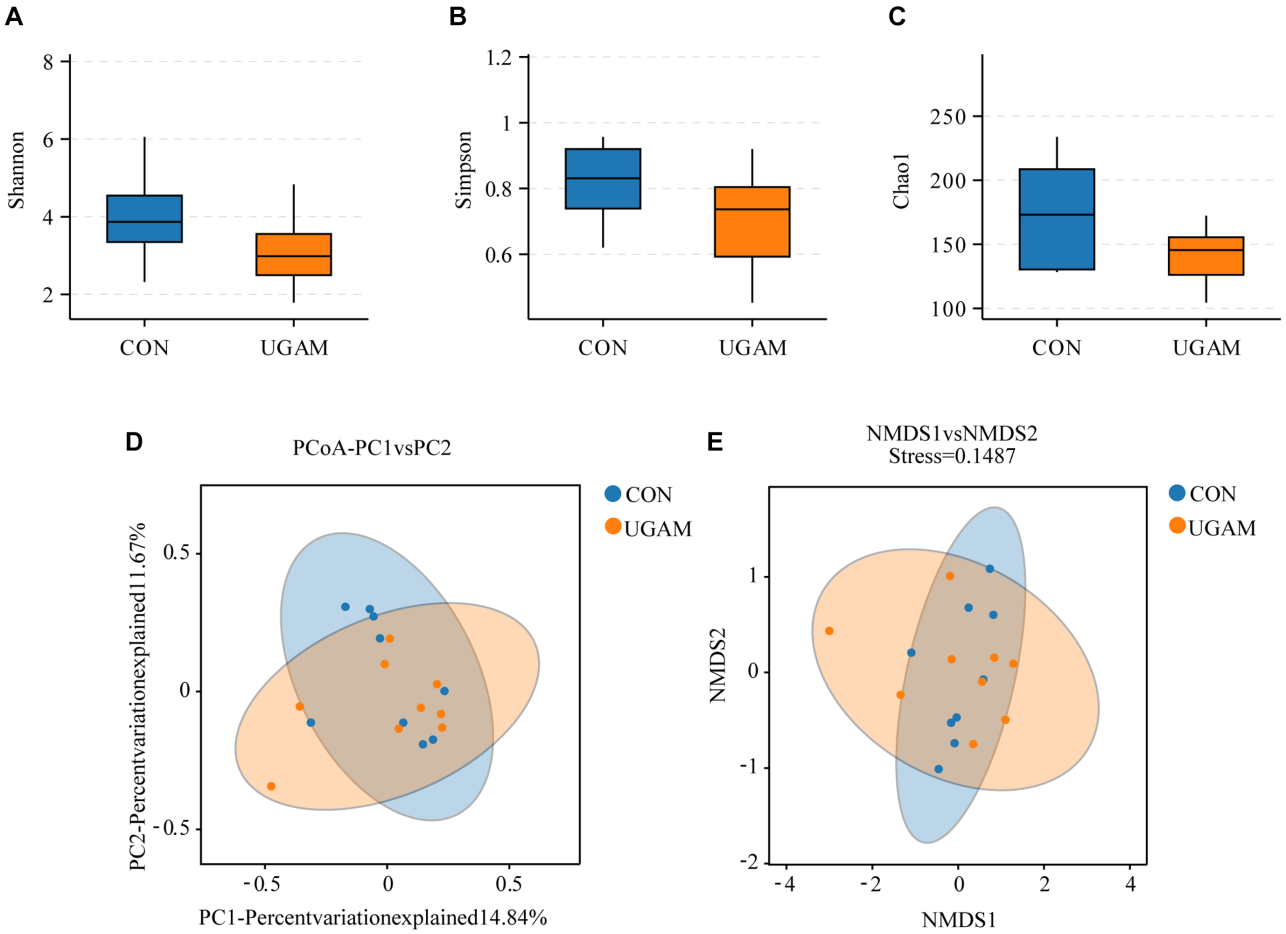
The alterations in the microbial richness and diversity of ileum chyme in goats fed the CON and UGAM diets. Summary of the Shannon **(A)**, Simpson **(B)** and Chao1 **(C)** indices in weaned goats. The principal coordinate analysis (PCoA; **D**) and Nonmetric Multidimensional Scaling (NMDS; **E**) of the ileum chyme-associated microbiota. CON, the control group; UGAM, the ultra-grinded *Astragalus membranaceus* powder group.

The effects of supplementing UGAM on the α-diversity of ileal microbiota were assessed by analyzing the Shannon, Simpson, and Chao1 indexes. Neither the Shannon and Simpson indexes nor the Chao1 index were affected by the UGAM supplementation (Figures 1A–C). Moreover, the inclusion of UGAM in the diet did not shift β-diversity as evidenced by the principal coordinate analysis (PCoA; Figure 1D) and Nonmetric Multidimensional Scaling (NMDS; Figure 1E) analysis, which showed no clear separation between the two treatments.

**Figure 2:**
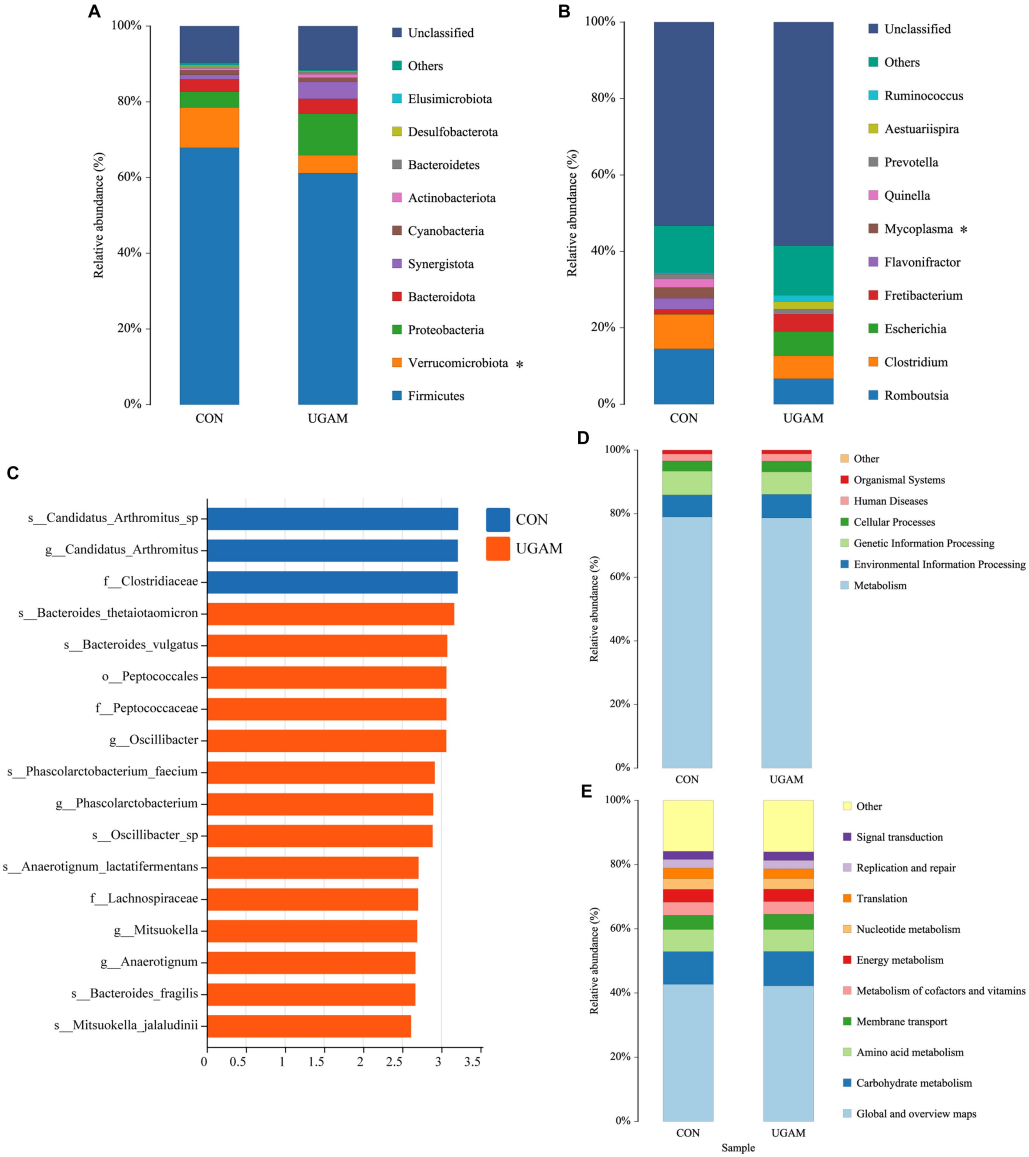
Results of 16S rRNA gene sequencing of the ileum bacteria of goats fed CON and UGAM diets. Taxonomic analysis of ileum bacteria of goats at phylum **(A)** and genus **(B)** levels. * indicates a significant difference between the CON and UGAM groups at *p* < 0.05; LEfSe analysis Histogram of the LDA scores for differentially abundant genera **(C)**; KEGG function prediction at class1 **(D)**, class 2 **(E)** levels. CON, the control group; UGAM, the ultra-grinded *Astragalus membranaceus* powder group.

The taxonomic compositions of the ileal microbes at different levels were analyzed, and the results showed that a total of 16 phyla, 23 classes, 57 orders, 92 families, 147 genera, and 186 species were detected in all samples from the two treatments. At the phylum level (Figure 2A), phyla *Firmicutes*, *Verrucomicrobiota*, *Proteobacteria*, *Bacteroidota*, *Synergistota*, and *Cyanobacteria* had relative abundances exceeding 1%, and the *Firmicutes* exhibited the highest relative abundance in both CON (67.9%) and UGAM (59.8%) treatments. The supplementation of UGAM had a lower (*p* < 0.05) relative abundance of *Verrucomicrobiota* than the CON treatment. At the genus level, a large proportion of sequences ranging from 57.5% to 59.3% remained unclassified (Figure 2B). Among the genera identified in the ileum, *Romboutsia*, *Clostridium*, *Escherichia*, *Fretibacterium* were the most abundant, followed by *Flavonifractor*, *Mycoplasma*, *Quinella*, *Prevotella*, *Aestuariispira*, and *Ruminococcus*. The UGAM treatment showed a lower (*p* < 0.05) relative abundance of *Mycoplasma* than the CON. The LEfSe analysis further showed significantly enriched microbial taxa between the two treatments (Figure 2C). Notably, the genera *Oscillibacter, Phascolarctobacterium, Mitsuokella,* and *Anaerotignum* were enriched in the UGAM treatment, whereas, the genera *Candidatus_Arthromitus* was enriched in the CON.

The PICRUSt2 analysis was performed to predict the contribution of identified microbial communities from the KEGG pathways at both level 1 and level 2 (Figures 2D,E). The KEGG functional annotation of the ileum microbiota indicated that the primary function of the ileum microbiota at level 1 was metabolism; while the major function related to metabolism at level 2 included metabolism of energy, carbohydrate, amino acid and nucleotide, and metabolism of cofactors and vitamins, as well as membrane transport. The ileum microbes have also evolved in translation, replication, repair and signal transduction. However, no significant differences were observed in the major functions between the CON and UGAM treatments.

### 3.5 Correlation among ileal bacteria at the genus level, markers of immunity, tight junction, and plasma biochemical indices

**Figure 3:**
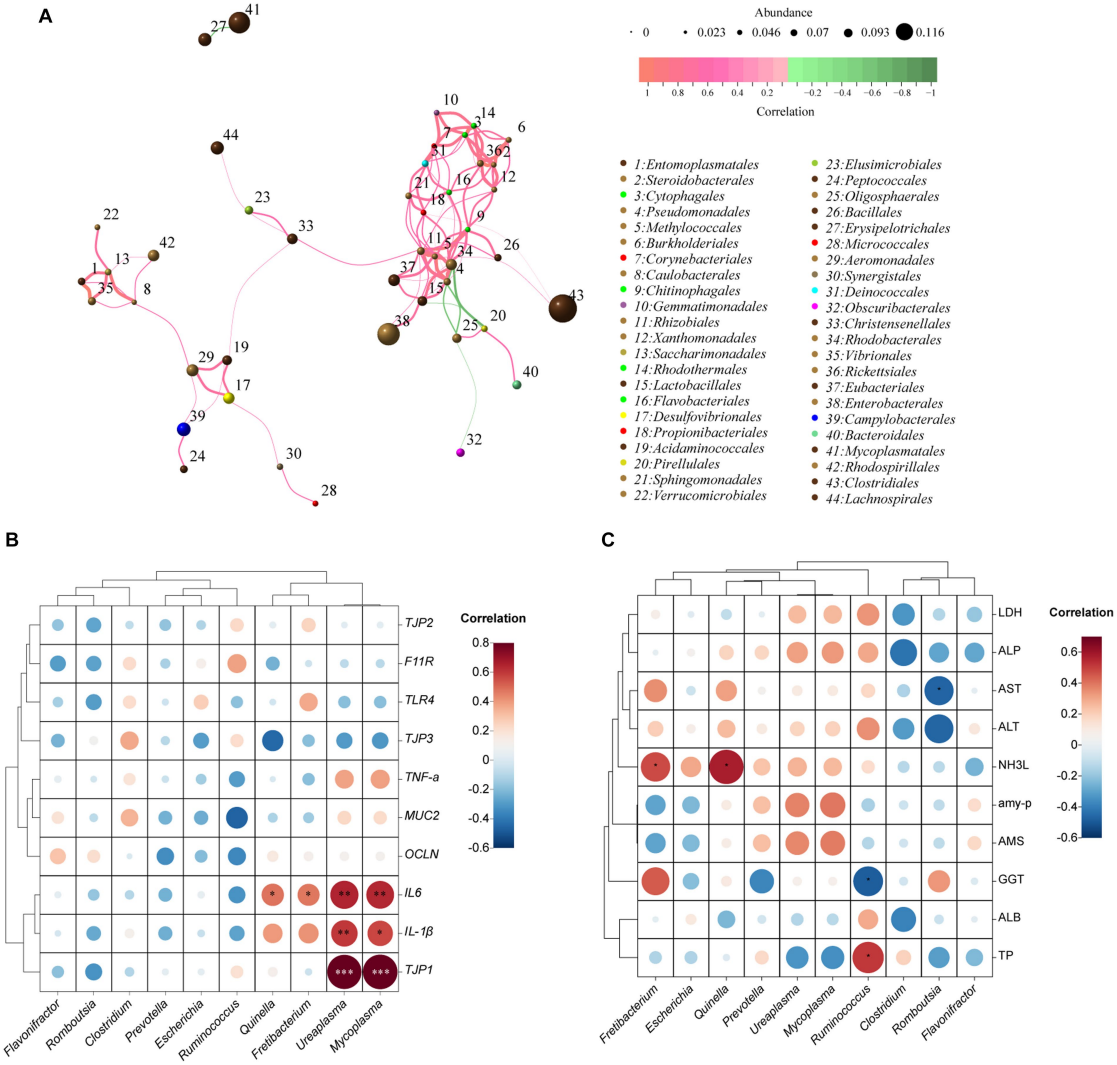
Correlation networks of the ileum bacteria at genus level, the relative expression of immune- and tight junction-related genes, and blood biochemical indices based on Spearman’s correlation coefficients. Network diagram at OUT level **(A)**. The size of the nodes indicates the mean abundance of each OTU. The lines between nodes represent correlations between the connected nodes, with the color saturation and line width indicating correlation magnitude: red denotes a positive correlation and green a negative correlation. Only lines corresponding to correlations with a magnitude greater than 0.1 are drawn; Heatmap illustrating the correlation between the relative abundance of the top 10 microbial genera with the relative expression of immune- and tight junction-related genes **(B)** and blood biochemical indicators **(C)**. The size and color of the circles represent the magnitude of the correlations, with larger and darker circles indicating stronger correlations. The red color indicates positive correlations, while the blue color indicates negative correlations.

The correlation network graph of the module (Figure 3A) depicts the interrelationships among 44 nodes representing 15 phyla, encompassing a total of 100 correlations. These correlations consist of 94 positive associations and 6 negative associations. Notably, *Rhizobiales* demonstrated the highest number (12) of positive correlations, whereas *Oligosphaerales* displayed the highest number (3) of negative correlations.

The correlation between ileal bacteria at the genus level and the relative expression of immune- and tight junction-related genes was presented in Figure 3B. The relative mRNA expression of *TJP1* (a tight junction-related gene) and *IL-1β* (an immune-related gene) exhibited a significant positive correlation with the relative abundances of *Ureaplasma* (R^2^ = 0.83, *p* < 0.001; R^2^ = 0.60, *p* < 0.01, respectively) and *Mycoplasma* (R^2^ = 0.84, *p* < 0.001; R^2^ = 0.57, *p* < 0.05, respectively). Similarly, the relative mRNA expression of *IL-6* demonstrated a positive correlation (*p* < 0.01) with *Ureaplasma* (R^2^ = 0.65) and *Mycoplasma* (R^2^ = 0.64), and a medium correlation (*p* < 0.05) with *Quinella* (R^2^ = 0.48) and *Fretibacteriun* (R^2^ = 0.47).

The correlation between ileal bacteria at the genus level and plasma biochemical indices was revealed in Figure 3C. The relative abundance of *Ruminococcus* showed a medium correlation (R^2^ = 0.52; *p* < 0.05) with TP, whereas had a negative correlation (R^2^ = −0.49; *p* < 0.05) with GGT. The abundance of *Fretibacteriun* (R^2^ = 0.48) and *Quinella* (R^2^ = 0.58) had a positive correlation (*p* < 0.05) with NH_3_L. Furthermore, the abundance of *Romboutsia* had a negative correlation (R^2^ = −0.47; *p* < 0.05) with AST.

## 4 Discussion

In recent years, there has been growing interest in studying the effects of natural plant extracts on nutrient digestibility, immune response, and microbiota composition in the intestines of animals because of their critical role in nutrient absorption and animal health. Additionally, a number of mechanisms whereby plant bioactive compounds may improve gut health, intestinal microbial balance and nutrient digestibility have been proposed; whereas, there are still very limited studies on this field because of the difficulty to obtain samples from the intestine and the high cost of animal use. Within our knowledge, this is the first study to assess the effects of dietary supplementation of UGAM on the ileal microbial community, expression of genes associated with intestinal immunity and tight junctions. Analyzing the combination of the ileal microbial community, gene expression associated with intestinal immunity and blood biochemical indices response to UGAM supplementation provides a great opportunity to better understand the mode of action of UGAM under stressed conditions (weaning). It is reasonably believed that the generated information from the present study is helpful for identifying and developing newer and better antimicrobials from natural products. However, it should be noticed that the current experiment was not able to identify the amount of each individual active compound from UGAM that reached the intestine, and thus, to determine the exact mechanism of the UGAM components. Therefore, these limitations in this study should be considered in future research. In addition, some speculations are included to expand the discussion based on the present results and the available findings in the literature, which should be interpreted with caution.

### 4.1 Effect of UGAM on blood biochemical indices of weaned goats

Blood biochemical indices serve as markers for the health and internal metabolic status of animals. Previous studies reported that dietary supplementation of *A. membranaceus* root powder increased activities of T-SOD, CAT, and T-AOC in the blood of weaned lamb (Zhong et al., 2012) and finishing lamb (Hao et al., 2020), suggesting enhanced antioxidant status. Although the antioxidant was not detected in the present study, a notable increase in blood TP and ALB contents with supplementation of UGAM, highlights an enhanced protein status within the body. The noteworthy elevation of TP may imply that the bioactive constituents present in UGAM aid in stimulating protein synthesis and metabolism. The ALB, predominantly synthesized in the liver, plays a pivotal role in the regulation of plasma osmotic pressure, maintenance of acid–base equilibrium, and facilitation of hormone transportation (Tothova et al., 2016). The ALP and GGT are markers for liver functionality, and abnormally elevated activities of these enzymes suggest potential liver impairment. Under the current experimental condition, the similar serum ALP and GGT activities between CON and UGAM treatments indicated that supplementation of UGAM at a daily dose of 10 g/head had no detrimental effect on the liver of weaned goats. The lack of differences in plasma indices between CON and UGAM treatments is in agreement with Zhong et al. (2012), who reported no effects of *Astragalus* polysaccharide and *A. membranaceus* root feeding on blood plasma metabolites such as blood urea N, high- and low-density lipoprotein–cholesterol, triglyceride, glucose, which are reflective of animal metabolism.

### 4.2 Effects on the ileal microbial community and functions

The gut microbiota assumes a substantial role in the host’s immune response and disease resistance. It contributes to intestinal health equilibrium through various mechanisms, including immune modulation, improved nutrient absorption, antimicrobial functions, and the preservation of intestinal barrier integrity (Ghosh et al., 2021). Several studies have demonstrated that *A. membranaceus* could regulate the gut microbiota of mice (Wang et al., 2023), broilers (Qiao et al., 2022), and pigs (Che et al., 2019) to maintain intestinal flora homeostasis and enhance intestinal health. In the current study, the CON and the UGAM groups had similar ileum microbial communities, suggesting that UGAM inclusion had little effect on modulating microbiota in the ileum of goats. Therefore, there were no significant differences in the major functions between the CON and UGAM treatments from the KEGG pathways at both level 1 and level 2. The failure to detect the effect of UGAM on ileal microbiota modulation is unclear. It may be speculated that some of the bioactive compounds in *A. membranaceus* were degraded in the rumen. It was reported that bio-activators, especially polysaccharides, in Chinese herbs and other plants could be degraded by ruminal microorganisms (Liu et al., 2015). In addition, the ultra-grinding that increases surface contact with microbial enzyme, could increase the ruminal degradation of bioactive compounds in *A. membranaceus*. It is consistent with our previous work that focused on the physic-chemistry properties of environmental factors on the physicochemical characteristics of the ruminal microbiota (Liu et al., 2017a,b). Although the ruminal microbial community was not measured, Zhong et al. (2012) reported an alteration of the rumen fermentation pattern with more propionate production by feeding *Astragalus* polysaccharide and *A. membranaceus* root, suggesting that bioactive compounds in *A. membranaceus* could alter ruminal microbiota.

In the present study, *Firmicutes* was the predominant phylum, succeeded by *Verrucomicrobiota*, *Proteobacteria*, and *Bacteroidetes*, which is consistent with the report by Jia et al. (2022) that *Firmicutes*, *Actinobacteria*, *Verrucomicrobia*, and *Bacterioidetes* constituted the predominant phyla in the goat ileum. Dietary supplementation of UGAM altered the taxonomic composition of ileal microbiota, with notable reductions in the relative abundances of *Verrucomicrobiota* at the phylum level and *Mycoplasma* at the genus level in the present study. The reduction in *Verrucomicrobiota* abundance could potentially impact the preservation of the intestinal mucosal barrier and immune regulatory functions (Bamola et al., 2017). The diminished presence of *Mycoplasma* may play a role in alleviating inflammation and adverse responses, consequently fostering intestinal well-being (Khadka et al., 2014). The LEfSe analysis further showed enriched genera of *Oscillibacter* and *Mitsuokella* in the UGAM treatment. Bacteria from the genus *Oscillibacter* are known as butyrate-producing bacteria (Shirvani-Rad et al., 2023), while bacteria from the genus *Mitsuokella* usually play a role in acetate production (Etxeberria et al., 2015). In agreement with the current study, Wang et al. (2023) reported that *Bacillus subtilis*-fermented *A. membranaceus* increased the abundance of butyrate-producing bacteria in the gut of hyperuricemic mice (Wang et al., 2023). Therefore, the enrichment of *Oscillibacter* and *Mitsuokella* in UGAM treatment would promote the production of short-chain fatty acids (SCFAs) in the hindgut of goat. It is widely accepted that SCFAs, especially butyrate, not only can maintain the stability and integrity of the epithelial barrier by regulating the expression of TJP such as claudin-1 and Zonula Occludens-1 (ZO-1), but also have anti-inflammatory activity by downregulating inflammatory cytokines such as IL-1β, IL-6, and IL-8 or exerting a direct anti-inflammatory effect (Shirvani-Rad et al., 2023). Moreover, *Mitsuokella* spp. is also a major bacterium fermenting protein in the small intestine (Duarte and Kim, 2022), and its enrichment in UGAM may improve the utilization of rumen by-pass protein. Dietary supplementation of *A. membranaceus* stems and leaves (Che et al., 2019) and polysaccharides derived from *A. membranaceus* polysaccharides (Qiao et al., 2022) also had beneficial effects on enhancing the relative abundances of *Prevotella* and *Ruminococcus* in weaned pigs and broilers. Therefore, *Astragalus* root powder has the potential to modulate the equilibrium of the intestinal microbiota and promote the health of the hindgut by enriching the beneficial bacteria.

### 4.3 Effects on the ileal immune and barrier functions

It has been reported that *A. membranaceus* root and extracts possess immunomodulatory properties, such as promoting the secretion of secretory immunoglobulin, and regulating the expression of cytokines in both serum and gut mucosa (Zhong et al., 2012; Che et al., 2019; Qiao et al., 2022). In the present study, the indices of immune organs, including thymus, spleen, and lymph nodes (jejunal, ileal, colonic), were not affected by the dietary inclusion of UGAM, which might be due to the short experimental period to differentiate the organ weight change between treatments. The gastrointestinal tract is considered as the largest immune organ in the body, and it ubiquitously expresses genes encoding proinflammatory factors that play a pivotal role in initiating immune and inflammatory responses. This is confirmed in the present study by the detected expression of immune-related genes, mainly cytokines (*IL-1β*, *IL-6*, *IL-17*, *TNF-α*, and *TGF-β1*) and factors involved in inflammatory signaling pathways (*TLR4*, *NF-κB*) in the ileal mucosa. The observed down-regulated expression of the *IL-6* gene in the ileum of UGAM-supplemented goats is somewhat in agreement with other studies. Qiao et al. (2022) reported that feeding of polysaccharides derived from *A. membranaceus* decreased serum concentrations of *IL-6* in broilers; Wang et al. (2023) found that *Bacillus subtilis*-fermented *Astragalus membranaceus* significantly attenuated the levels of inflammatory factors *IL-6* in the kidneys of hyperuricemic mice. The *IL-6* prompts leukocyte adhesion and initiates complement system activation, resulting in microcirculatory obstruction and tissue cell impairment (Roumenina et al., 2016). The *IL-6* is induced often together with the proinflammatory cytokines *TNF-α* and *IL-1,* and circulating *IL-6* plays an important role in the anti-inflammatory. The observed decreased expression of the *IL-6* gene without treatment effect on other measured inflammatory factors suggests that supplementation of UGAM might have improved intestinal health and inflammatory response, and possibly inhibition of inflammation in the ileum of weaned goats.

The gut barrier, composed of an extracellular mucus layer and intestinal epithelial cells, plays an important role in protecting and maintaining gut health. Mucins secreted by goblet cells are an important component of the mucus layer, and act as the first line for the prevention of pathogen invasion (Grondin et al., 2020). Meanwhile, tight junctions of the small intestine act as the baseline for maintaining intestinal barrier integrity and ensuring normal barrier function (Zeisel et al., 2019). MUC-2 is involved in maintaining the thickness of the intestinal mucus layer, while the integral membrane proteins (such as occludin and claudins) and cytoplasmic plaque protein ZO-1 are the major components of tight junctions (Qiao et al., 2022). The effects of *A. membranaceus* root on the intestinal barrier function of ruminants have not been reported; meanwhile, dietary *Astragalus membranaceus* polysaccharides supplementation benefits the intestinal health of broilers by improving intestinal barrier function has been consistently reported. Wang et al. (2021) found that *Astragalus membranaceus* polysaccharides could elevate the mRNA expression of *OCLN* and *ZO-1* and the number of goblet cells in the jejunum of broilers. Qiao et al. (2022) reported that dietary supplementation of *Astragalus membranaceus* polysaccharides remarkably upregulated *OCLN*, *CLD1* and *MUC2* mRNA expression in the duodenum, jejunum, and ileum of broilers. In the current study, the mRNA expression of *MUC-2*, *OCLN*, *CLD1*, *TJP1*, *TJP2* and *TJP3* in the ileum of goats were determined, with lower expressions of *OCLN* but greater expression of *TJP3* observed in goats received UGAM. The lower expression of *OCLN* might lead to a thinner mucosal layer, whereas the greater expression of *TJP3* would improve the tight junction of ileal epithelial. These results appeared to be contradictory, and there was no clear explanation of it.

## 5 Conclusion

Supplementation of UGAM in weaned goats’ diet exerts anti-inflammatory activity, enhances plasma protein metabolism status, and has no evidence of detrimental effects on the liver and other organs. More importantly, feeding ultra-grinded *Astragalus* root powder to weaned goats has the potential to modulate the equilibrium of the intestinal microbiota and promote the health of hindgut by enriching the beneficial bacteria, especially the butyrate-producing bacteria.

## Data availability statement

The datasets presented in this study can be found in online repositories. The names of the repository/repositories and accession number(s) can be found below: NCBI SRA, PRJNA1030129.

## Ethics statement

All the protocols for the use of animal and experimental procedures in this study were approved by the Animal Care Committee according to the Animal Care and the Use Guidelines (CAS-ISA-2018-1250) of the Institute of Subtropical Agriculture, Chinese Academy of Sciences (Changsha, China). The studies were conducted in accordance with the local legislation and institutional requirements. Written informed consent was obtained from the owners for the participation of their animals in this study.

## Author contributions

GL: Writing – original draft, Data curation, Visualization, Formal analysis. KG: Writing – review & editing, Formal analysis. CZ: Writing – review & editing, Supervision. ZT: Writing – review & editing, Resources, Supervision, Validation. WY: Writing – review & editing, Supervision. DN: Writing – review & editing, Supervision. TR: Writing – review & editing, Data curation, Funding acquisition, Supervision. YL: Conceptualization, Data curation, Formal analysis, Funding acquisition, Investigation, Methodology, Project administration, Resources, Supervision, Visualization, Writing – review & editing.
